# Connection Changes in Somatosensory Cortex Induced by Different Doses of Propofol

**DOI:** 10.1371/journal.pone.0087829

**Published:** 2014-02-07

**Authors:** Zhaoduan Li, Xingkui Liu, Yi Zhang, Jinshan Shi, Yu Zhang, Peng Xie, Tian Yu

**Affiliations:** Department of Anesthesiology, Zunyi Medical College, Zunyi, Guizhou, People's Republic of China; Massachusetts General Hospital, United States of America

## Abstract

**Background:**

The mechanism by which general anesthetics, widely used in clinical practice for over 160 years, effects on sensory responsiveness has been unclear until now. In the present study, the authors sought to explore the effect of different doses of propofol on somatosensory cortex by whisker stimulation in rats.

**Methods:**

In a fixed cage, rats were anesthetized with propofol 80 mg/kg intraperitoneally and then cathetered tail vein with 23-gauge metal needle connected with a pump. Two holes (2 mm diameter) were drilled and recording electrodes implantated in the primary somatosensory cortex barrel field (S1BF) and secondary somatosensory cortex (S2). The extracellular (20 rats) and intracellular (8 rats) recordings were used to test the neuron activity in both cortices at different doses of propofol (20, 40 and 80 mg/kg/h) through tail vein by pump. Meantime, vibrissal, olfactory, corneal responses (VOCR, sedation), and tail-pinch response (TRP, analgesia) were tested every 10 min during the doses of propofol 20, 40 and 80 mg/kg/h.

**Results:**

VOCR and TRP were depressed by propofol in a dose-dependent manner. The amplitude by whisker stimulation in S1BF was stronger and the peak latency was shorter compared with that of in S2. The response latency of S1BF and S2 was increased by raising infusion rate of propofol with the response latency in S2 being longer than that in S1BF at the same doses of propofol. The cross-correlation between S1BF and S2 decreased as the propofol infusion rate increased. The input resistance was higher by increasing infusion rate of propofol.

**Conclusion:**

The sedation and analgesia effects of propofol were dose-dependent. Both the connectivity and instinctive oscillation between S1BF and S2 were proportionally modulated by the different doses of propofol.

## Introduction

General anesthesia has been used in clinical practice for more than 160 years, but the neurophysiological mechanisms of unconsciousness, amnesia, analgesia and immobility are still unclear, especially for the mysterious unconsciousness. Scientific evidence suggests that various agents affect different, well-described regions of the central nervous system by acting on different receptors and receptor subunits [1], [2]_,_ leading to highly agent-specific effects. Such different targets may result in various clinical effects of different anesthetics.

Due to its high temporal resolution, compared with other neuroscientific techniques, the electrophysiology technique has been a major focus of medical research by neuroscientists for decades. Some investigators concluded that particular anesthetic has an effect on the evoked response and it is important to consider the agent-specific mechanisms that drive general anesthesia [3], [4]. Several investigators have found differences of evoked potential in latency and amplitude under various anesthetic conditions with the use of in vivo and in vitro approaches on different physiological systems [5], [6], but no research in vivo between primary somatosensory cortex barrel field (S1BF) and secondary somatosensory cortex (S2) of propofol and behavioral condition has been performed.

Rats can distinguish between objects that differ only in micron-scale surface texture by their whiskers [7]. This relies on the signal processing in barrel cortex [8], therefore the rodent whisker sensory system is a powerful model for information processing in the cortex. Somatosensory cortex in the rat is typically divided into primary somatosensory cortex (S1) and secondary somatosensory regions (S2). The barrel field of S1 (S1BF) receives dominant projections from the special relay nuclei from the thalamus with the S2 region reciprocally connected to S1B, responding at longer latencies to afferent input compared with S1BF. S1BF in the rat forms a single somatotopically organized representation of the body with the hindquarters pointed medially, the limbs rostrally, and the facial representation dominated by the barrel cortex [6], [9]–[11]. However, the somatotopic organization of secondary somatosensory regions (S2) and whether the relationship between S1BF and S2 is parellel or hierarchical is still unclear; although extensive studies have focused on the thalamocortical and corticothalamic neuron network system [12]. Furthermore, we still do not know the effect of various anesthetics such as the common clinical intravenous anesthetic propofol on sensory input of the somatosensory including S1BF and S2.

The aim of this study was to determine the effects of three different doses of propofol on the electroencephalographic activity on the somatosensory cortex. The effect of different doses of propofol on sensory input between S1BF and S2 by whisker stimulation was investigated in our study. We hypothesized that propofol would produce a gradual electroencephalographic synchronization effect with using propofol 20, 40 and 80 mg/kg/h, correlating with suppression of animal behavior as well as sensory input in somatosensory cortex. We expected that the observed differential effects of propofol would, in the long run, provide insights into propofol specific mechanisms of anesthesia and help us better understand the general anesthesia mechanism.

## Materials and Methods

### Animals

Sprague Dawley rats (250–300 g) were kept in the constant temperature (22±2°C, 30% humidity), 12-h light/dark-controlled at the Animal Facilities of Chinese Academy of Science at Shanghai, China, with free access to rodent chow and water. Animal care was approved in accordance with the guidelines for Care and Use of Laboratory Animals in China.

### Experimental Procedure

Rats were fixed in a cage and anesthetized with propofol (CA742, AstraZeneca UK Limited) 80 mg/kg intraperitoneally, then cathetered tail vein with 23-gauge metal needle when the rats lost righting reflex. All the rats were placed in a stereotaxic frame, a ceramic pressure sensor was used to monitor respiration and heart rate. A rectal temperature probe was maintained at 37°C with a thermostat-controlled heating pad. A tube filled with oxygen continuously fixed in front of nose in order to prevent hypoxia. The skin was laterally reflected, and the cranium was gently scraped of connective issue and bleeding was cauterized. Two holes (2 mm diameter) was drilled in the skull above the implantation of recording electrodes including S1BF (barrel cortex) and S2 in right hemisphere. The S1BF was as follows: flat skull, bregma origin, anterior −2 mm, lateral 5.8 mm, and S2: flat skull, bregma origin, anterior −1.8 mm, lateral 6.5 mm (The Rat Brain in Stereotaxic,Paxinos and Watson 1986). All experimental procedures were approved by the Institutional Animal Use and Care Committee of Chinese Academia of China at Shanghai.

The initial propofol infusion rate was 20 mg/kg/h by pump and then increased to 40 mg/kg/h and later 80 mg/kg/h. During all the experiments, the rats were allowed to breathe pure oxygen spontaneously. Two tungsten microelectrodes (impedance 450 KΩ) were slowly lowered into the S1BF and S2 with micromanipulator for extracellular recordings, meanwhile two reference electrodes were fixed below the scalp for 20 rats. Intracellular recordings in S1BF were performed by sharp glass microelectrodes for another 8 rats. Both extracellular recording and intracellular recording were recorded during propofol 20,40 and 80 mg/kg/h. Recordings were performed for 20 min (10 min spontaneous state and 10 min whisker stimulation). The impedance of the electrode (filled with 2 M potassium acetate) was 60–100 MΩ. The electrode was slowly advanced under a dissecting microscope. The discharge properties of neurons in S1BF were recorded and the input resistances corresponding to different doses of propofol with or without left whisker stimulation were tested by 200 pA (duration 100 ms) as input current in neuron. Meantime, for evaluating the anesthesia depth in rates, tail-pinch response (TPR) and vibrissal, olfactory, and corneal responses (VOCRs) were to test the reaction of rats [13]. At the conclusion of the experiment, animals were killed by overdose anesthesia without consciousness. In addition, blood was collected and measured for blood gas analysis from 20 rats for extracellular recordings and 8 rats for intracellular recordings during the infusion rate of 20,40, 80 mg/kg/h. The neuron activities of S1BF and S2 were recorded simultaneously and for convenience, group A, group B and group C were named to compare the changes between different doses of propofol 20, 40, 80 mg/kg/h accordingly.

### Data Analysis

Raw signals were visually inspected and artifacts were deleted before analysis. MATLAB (Mathworks,), OriginLab 8 (OriginLab) and Spike 2 software were used for data analysis. We calculated the integrations of EEG power within the following bands: total, 1–100 Hz; δ, 1–4 Hz; θ, 4–8 Hz; α, 8–12 Hz; β, 12–25 Hz; γ, 25–100 Hz, spindle wave 7–14 Hz. The extracellular signal was extracted to compare the MU (multi-unit) data through a band-pass filter (600–3000 Hz) for a more specific description. The uniformity of the spike train is described with the absence and the presence of whisker stimulation at different doses of propofol. Meanwhile, the somatosensory-related potentials (SEP) parameters (amplitude, peak latency and response latency) and connectivity between S1BF and S2 at different doses of propofol anesthesia by whisker stimulation were calculated by Spike 2 software.

For intracellular recordings, we first distinguished the Up and Down states by comparing the average membrane potential during a given time window (∼10 ms) with that of a much larger time span (∼3 s). We then counted the frequency of membrane potential transitions between the two states and used Ohm’s law R = U/I to calculate the input resistance with the 200 pA input current. Up and Down states: To describe the uniformity of the spikes, we defined a “Non-uniform Index” of any given time period with a spike number of as
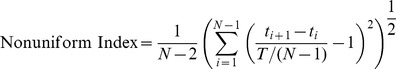
where *t_1_* stands for the time of the *i* th spike. This index adequately caught the distinct characteristics of the “Up and Down” in contrast to the persistent “Up” firing patterns, regardless of their overall mean firing rates.

### Statistical Analysis

Continuous data were given as mean ± standard deviation (SD) if distributed normally, or median (10^th^–90^th^ percentiles) if not distributed normally. Analysis of variance (ANOVA) was used to test for a significant difference in observations at different doses of propofol. Comparison between the same group at the same state was carried out using a paired t-test. For the test, p<0.05 was accepted for statistical significance.

## Results

### Behavioral Responses to Sensory Stimulation

Table 1 shows dose-dependent group-average effects with the different doses of propofol. We want to compare the sedation and the analgesia effect of propofol simultaneously, so we combined vibrissal, olfactory and corneal responses to form a single index (VOCR) except tail pinch response (TPR) [13]. When the rats were infused with propofol 20 mg/kg/h, all the responses except vibrissal remained the same. As the infusion rate was increased to 40 mg/kg/h, no response was detected to vibrissal stroking compared with 20 mg/kg/h. When infusion rate was increased to 80 mg/kg/h, only observed little response to tail pinch. It showed that there was no significant change in the index of blood gas, glucose or hematocrit, and there is also no significant influence in heart rate, while respiratory rate was lightly depressed (table 2). For convenience, group A, group B and group C were named to compare the changes between different doses of propofol 20, 40 and 80 mg/kg/h accordingly.

**Table 1:** Dose-dependent group-average effects of propofol on VOCR and TPR.

Group	VOCR	TPR
A	3.5±0.6	1.9±0.5
B	1.2±0.5*	1.1±0.4*
C	0.5±0.4*▴	0.5±0.4*▴

Compared with group A and group B,*p<0.05 and ▴p<0.05 mean level of significance, respectively.

**Table 2:** Blood gas values, Heart rate and Respiratory rate.

Parameters	group A	group B	group C
pH	7.31±0.02	7.30±0.02	7.28±0.03
PaCO2 (mmHg)	43±2	45±3	47±3
PaO2 (mmHg)	98±3	98±4	97±4
SpO2	98.8±0.6	97.9±0.5	97.5±0.7
Base excess (mmol/L)	−5.3±0.9	−5.0±0.8	−4.6±1.0
Glucose (g/dL)	15.1±0.6	13.4±0.7	14.3±0.4
Hematocrit (%)	48.3±1.4	46.2±1.5	45.9±1.2
Heart rate(min-1)	348±64	301±56	268±51
Respiratoryrate(min-1)	129±12	102±11	81±9

### EEG Power in S1BF and S2

**Figure 1:**
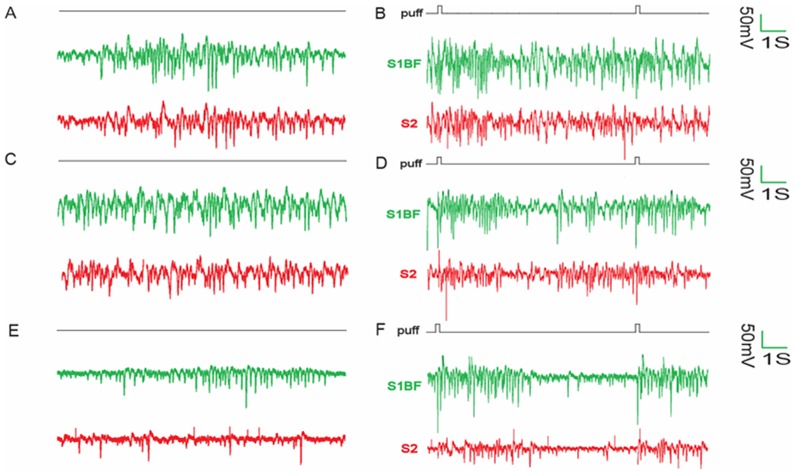
Simultaneous extracellular recordings in S1BF and S2 by different doses of propofol. Spontaneous activity (A, C, E) and whisker-triggered responses (B, D, F) were shown in different panels. Extracellular recording were performed in S1BF (green) and S2 (red). Panel A and B: 20 mg/kg/h; Panel C and D: 40 mg/kg/h; Panel E and F: 80 mg/kg/h.

**Figure 2:**
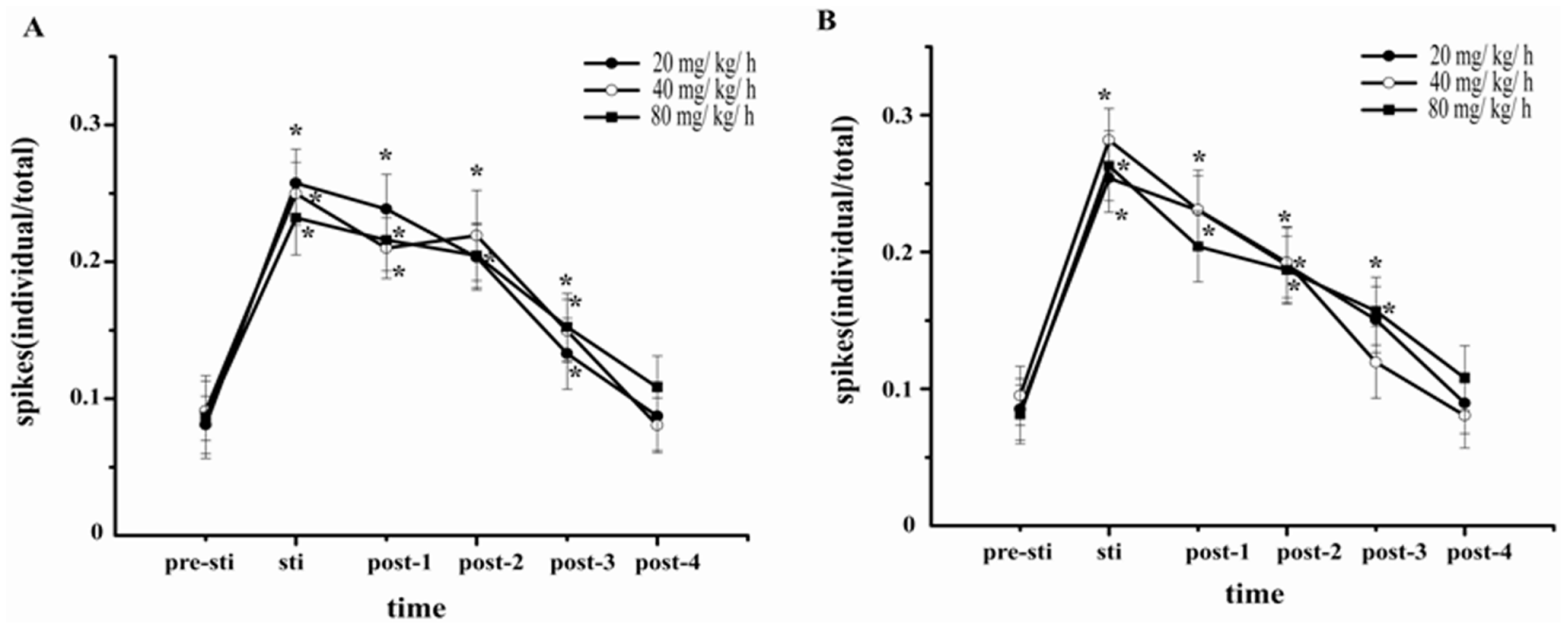
The spikes by whisker stimulation in S1BF and S2 by different doses of propofol. The response in S1BF (A) and in S2 (B). The change in spikes was expressed along different time period since the onset of drug application. The spikes triggered by whisker stimuli were significantly increased compared with pre-sti. It gradually decreased to the basic level (pre-sti) in 4 s. (pre-sti: 1 s before stimuli; sti: stimulation; post-1∶1 s after stimulation; post-2∶2 s after stimulation; post-3∶3 s after stimulation; post-4∶4 s after stimulation).

**Figure 3:**
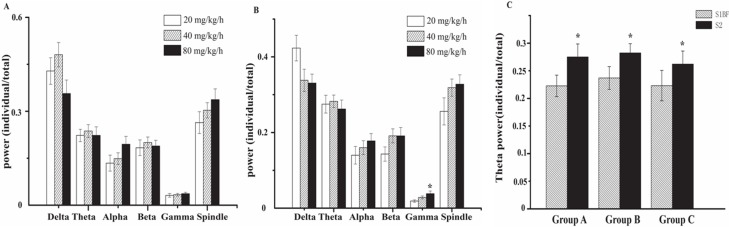
The changes of EEG power: Delta (1–4 Hz), Theta (4–8 Hz), Alpha (8–12 Hz), Bata (12–25 Hz) and Gamma band(25–100 Hz) as well as Spindle wave(7–14 Hz) in S1BF and S2 when the rats were infused with propofol infused at 20, 40 and 80 mg/kg/h. A) EEG power in S1BF at different dosage of propofol and no difference among the three groups(infusion rates); B) EEG power in S2 at different dosages of propofol and no difference among the three groups except the delta power in propofol 80 mg/kg/h was stronger than that in propofol 20 mg/kg/h; C) The change of theta power under different dosages of propofol. The power of θ wave in S2 was larger than that in S1BF In any group A,B and C *p<0.05.

Fig. 1 showed the raw recordings of simultaneous extracellular recordings in S1BF and S2 at different doses of propofol by or not by left whisker stimulation. Firstly, we observed that S1BF and S2 produce a train of spindle wave with or without whisker stimulation. Observing for a more clear tendency, spikes (multi-unit data) were compared in S1BF and S2 (Fig. 2) illustrating that the number of spikes dramatically increased after whisker stimulation compared with pre-stimulation (*p*<0.05). It then gradually decreased to baseline within ∼4 s (Fig. 2). Significant increase was only detected in gamma wave in S2 while the infusion rate was increased from 40 to 80 (Fig. 3 A–B) during EEG power analysis. Subsequent analysis found that the power of theta wave in S2 was larger than that in S1BF (Fig. 3.C).

### The ERP Properties and Cross-correlation between S1BF and S2

**Figure 4:**
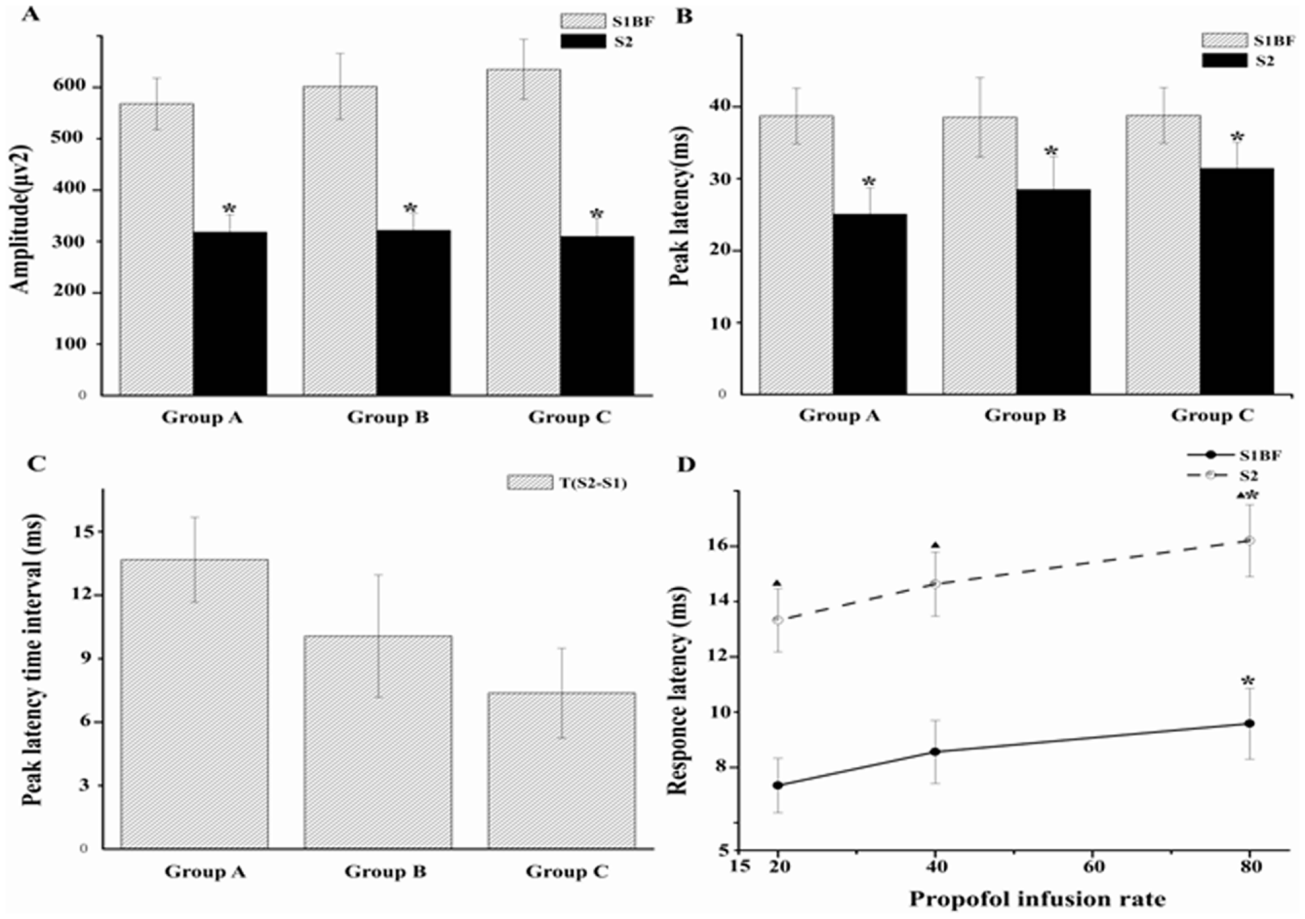
The ERP amplitude and latency (peak and response latency) in S1BF and S2. Panel. A) The left, upper graph shows the change of amplitude by whisker stimulation. The amplitude of S1BF was larger than that in S2 in group A, B and C. B) The right, upper graph shows the change of peak latency by whisker stimulation. The peak latency of S1BF was longer than that in S2 in group A, B and C. C) The left, lower graph shows the time interval of peak latency between S1BF and S2 and there was no difference among the three groups. D) The right lower graph shows the response latency in S1BF and S2. The response latency of S1BF was shorter than that in S2 in three different rates. Compared with group A(20 mg/kg/h), the response latency in group C(80 mg/kg/h) was longer in both S1BF and S2. It gradually decreased to the basic level ▴ p<0.05, *p<0.05, level of significance.

**Figure 5:**
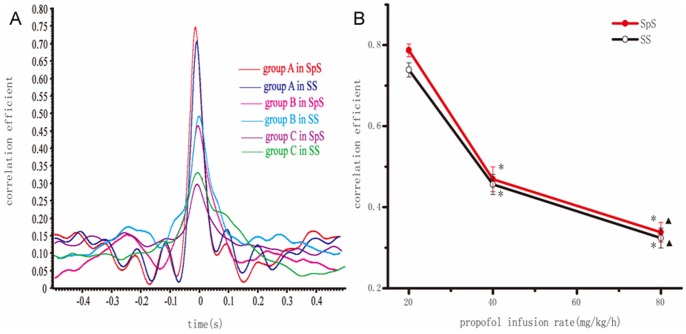
The cross-correlation between S1BF and S2 at different doses of propofol. A) The correlation coefficient between S1BF and S2. B) The averaged correlation coefficient was calculated with different infusion rate of propofol. The correlation coefficient was decreased gradually as raising the doses of propofol *p<0.05 and ▴p<0.05. SpS: spontaneous state, SS: stimulation state.

The amplitude and peak latency of event-related potentials (ERP) in S1BF were significantly larger and shorter than that in S2, respectively (*p*<0.05, Fig. 4.A. and Fig. 4B). The response latency in S1BF was shorter than that of S2 (Fig. 4.D). In group C (80 mg/kg/h), the response latency was longer than that in group A (20 mg/kg/h, *p*<0.05, Fig. 4.D). As the propofol infusion rate increased, the connectivity between S1BF and S2 dose-dependently decreased (Fig. 5.A), cross-correlation coefficients in spontaneous or stimulation state were decreased significantly (*p*<0.05, Fig. 5.B).

### The Intracellular Recording in S1BF at Different Doses of Propofol

**Figure 6:**
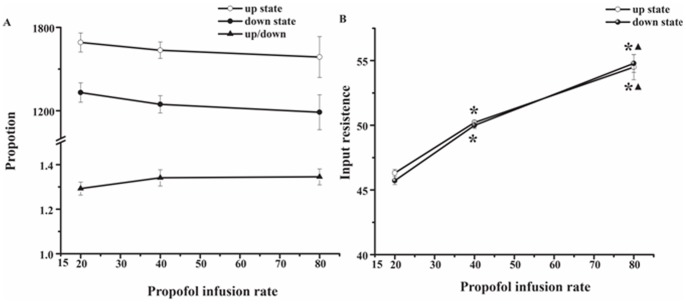
The up and down state in S1BF at different doses of propofol. A) The proportion of up state, down state and up/down state in S1BF and found no significant difference between the three groups. B) The relationship between the input resistance and different infusion rate. Compared with groups, the input resistance was larger than that in group A (20 mg/kg/h) and smaller than that in group C (80 mg/kg/h) *p<0.05 and ▴p<0.05.

It showed that propofol had no effect on the up state and down state (Fig. 6A), while the input resistance increased with increasing doses of propofol (Fig. 6B).

## Discussion

### Spindle Wave, Thalamus and Cortex

Thalamus is a major “gateway” in corticothalamocortical functional connections including consciousness and cognition, such as learning and memory, as well as sleep-wake cycles [14]. Functional imaging studies in both humans and other animals have showed that direct and indirect depression of thalamocortical neurons provide a convergent point for neural pathways of anesthetic action leading to a sleep-like state [15]. Mutually interconnections among cortical, nRT, and thalamic relay neurons exhibit phasic behaviors such as tonic and burst firing that represent different functional modes [16]. Due to some difficulties in identifying the beginning of spindle wave, spindle wave was not analyzed. When we compared with the shape of spindle wave reported during sleep cycle, we found that most of them were not significantly different. So we may assume that propofol triggered the spindle wave by activating GABA_A_ receptor through the thalamus-cortical pathway, meanwhile the propofol could generate the anesthesia effect which is mostly similar to natural sleep because of the same spindle wave from microscopic observation. In other words, we have reason to believe that propofol performs “natural anesthesia” on the human body with less side effects compared to other anesthetics.

### Event-related Behavioral Responses

The VOCR and TPR were depressed by propofol in a dose-dependent manner and the result is consistent with Narimatsu E’S [5], which showed that the sedation and analgesia effect of propofol were dose-dependently and significantly different as we increased the doses of propofol. Until now, Pashkov et al. [3] confirmed that propofol exerts its antinociceptive effect through inhibiting dorsal root neurons in the spinal cord while administered by intravenous route. Propofol administered intrathecally in rats has also been shown to produce antinociception [3], [4]. We observed dose-dependent changes in behavior and found propofol depressed VOCR and TPR significantly in a dose-dependent manner, which means propofol dose-dependently caused sedative and analgesia effect. It is consistent with the notion that propofol iv probably modulates the central sedation or descending antinociceptive systems in the periaqueductal gray matter-the source of descending inhibitory control of spinal nociceptive inputs [17], [18] and other possible targets, such as medial thalamic nuclei [19] and caudate-putamen [18]. It was found that the analgesic effect of propofol may result as it potentiate the functions of GABAA receptors [20]. Our results were in agreement with Antognini et al. [21] who found propofol directly repress the lumbar dorsal horn neuronal responses to noxious stimulation when goats were infused with propofol intravenously. However, Jugovac [13] found that propofol could not decrease the TPR score Intracerebroventricularly, indicating that propofol has no analgesia effect. The disparity indicates the drug administration route plays an important role in research. In all, it indicated that the sedation and analgesia effect of propofol may occur at different brain regions. That is, the former sedative effect was induced by regulation of cerebrum and the later analgesia with no response to nociceptive stimulation, might be accomplished by modulation of spinal cord [22].

### EEG and SEP

We firstly observed that S1BF and S2 produce a train of spindle wave with or without whisker stimulation (due to technology difficulty in S2 recording, we have no intracellular recording in S2). To observe more clearly, we compared the multi-unit data of extracellular recording in S1BF and S2 and found that the number of spikes dramatically increased after whisker stimulation compared with pre-stimulation. For EEG power, we only found an increased gamma wave in S2 as the infusion rate of propofol increased. According to a popular 20-year-old theory, gamma waves may play an important role in integrating the unity of conscious perception [23]. Ma et al. [24] also found that an increase and decrease of 30–50 Hz (gamma) waves in the hippocampus correlated with behavioral activity anesthetized by halothane and isoflurane, respectively. Although it is still unknown whether gamma wave activity is related to subjective awareness at the present time, we found gamma wave increased as the infusion rate of propofol increased in S2 but not in S1BF. This indicates different asynchronized effect on different brain regions even in the same anesthesia depth. The gradually increasing effect of propofol on gamma wave correlated well with different sedation levels.

We observed that there was no difference in the amplitude and peak latency of whisker triggered responses. However, the amplitude caused by whisker in S1BF was larger than that in S2, while the peak latency was shorter than that in S2 at the same infusion rate. There was no difference found between S1BF and S2 in different anesthesia depth found no difference. In S1BF, the response latency in light anesthesia (20 mg/kg/h) was shorter than that in deep anesthesia (80 mg/kg/h) and response latency was longer than that in S1BF at any infusion rate.

### The Co-activation between Thalamus, S1BF and S2

From the experiment, we observed that the response latency changed across different doses of propofol, but peak latency did not. At the same dose, the amplitude and peak latency of S1BF were larger and shorter than that in S2, respectively. Moreover, the higher the infusion rate, the longer the response latency in both S1BF and S2. Our results were consistent with several previous studies [25], [26], which examined epipial-evoked potentials by stimulating one whisker and other body regions, and found a much longer latency of evoked response in S2 than that in S1BF. However other studies showed that S1BF and S2 neurons responded to peripheral stimulation with similar peak latencies in rabbits. Kwegyir-Afful et al. [27] obtained extracellular unit recordings from narcotized rats, in response to whisker deflections triggered by a piezoelectric device, and compared response properties of neurons in S1BF (layer IV) and S2 (layers II to VI), finding that neurons in both areas have similar response latencies. However there are some significant differences in the neuronal properties of S1BF and S2. Furthermore, S1BF fast-spiking (inhibitory) and regular spiking (excitatory) units had different spontaneous and evoked activity levels and differed in their responses to stimulus onset and offset. However, S2 neurons did not show significant differences in these properties. Kwegyir-Afful et al. [27] interpreted the properties of S2 neurons by a hypothesis that S2 neurons are driven by thalamic inputs and are a part of paralemniscal system. Thus whisker-related inputs are processed in parallel by a lemniscal system involving S1BF and a paralemniscal system that processes complimentary aspects of somatosensation and S2 is parallel with S1BF but not hierachical. The discrepancy between S1 and S2 may be due to variations in stimulation modality or recording methods. In the current study, mechanical whisker stimulation was applied, while studies by Kwegyir-Afful [27] and Liao [28] used electrical stimulation applied to the forepaw. The effect of recording methods or stimulation modalities on measurements of response latencies needs to be further investigated.

Lin et al. [29] used electron microscope to study the mode of termination of cortico-thalamic fibers and showed a hierarchical scheme of somatosensory transmission pattern, in which peripheral information was processed sequentially from ventral posterior complex thalamus to S1BF and then to S2 orderly. This hypothesis was supported further by lesion studies, that is, removal of an S1BF representation and found no somatic evoked response in the corresponding S2 region in macaques and rhesus monkeys [30]–[32]. In contrast, S1BF responsiveness was not affected by the abolished homotypical S2 region [31]. Electrical studies further supported the notion that S1BF projects sensory information to S2 because S1BF neurons responded to peripheral stimulation like whisker-stimulation at a shorter response latency than S2 neurons in rats [25], [26], [33], which is consistent with ours. We found the cross-correlation between S1BF and S2 decreased with increasing infusion rate, which indicated that not only the connectivity between S1BF and S2 were affected by the different doses of propofol, but also the information processing between S1BF and S2 was interrupted or unsyncronized at different doses of propofol. Sheeba [34] introduced a thalamocortical model of interacting neuronal ensembles to describe phase relationships, which implied that the neuronal ensembles inhibit information coding during deep anesthesia and facilitate it during light anesthesia. The cross-correlation coefficient between S1BF and S2 decrease as increasing the propofol infusion rate in spontaneous level or in stimulation level. Intracellular recordings illustrated that propofol did not affect the Up and Down state in the single neuron. However the input resistance was higher as we increased the doses of propofol. It suggested that propofol could block some ion channels such as L-type calcium [35], which slowed down membrane potentials. Propofol, a GABAA receptor activation and modulation, enhances receptor function by increasing the probability of opening ion channel and drug actions on ligand-gated ion channels, which may be the reason for unconsciousness caused by propofol [36].

In conclusion, the sedation and analgesia effect of propofol are dose-dependent. Both the connectivity between S1BF and S2 and the instinctive oscillation are affected by different doses of propofol. The present study provides another piece of evidence in support of the hierarchical processing of somatosensory flow by the S1BF and S2 in the rats.
